# Homeobox gene *Rhox5 *is regulated by epigenetic mechanisms in cancer and stem cells and promotes cancer growth

**DOI:** 10.1186/1476-4598-10-63

**Published:** 2011-05-24

**Authors:** Qiang Li, Mark E O'Malley, David L Bartlett, Z Sheng Guo

**Affiliations:** 1 The University of Pittsburgh Cancer Institute, University of Pittsburgh, Pennsylvania 15213, USA; 2 Department of Surgery, University of Pittsburgh School of Medicine, Pittsburgh, Pennsylvania 15213, USA

## Abstract

**Background:**

Homeobox genes murine *Rhox5 *and human *RHOXF1 *are expressed in early embryonic stages and then mostly restricted to germline tissues in normal adult, yet they are aberrantly expressed in cancer cells *in vitro *and *in vivo *. Here we study the epigenetic regulation and potential functions of *Rhox5 *gene.

**Findings:**

In *Rhox5 *-silenced or extremely low expresser cells, we observed low levels of active histone epigenetic marks (H3ac, H4ac and H3K4me2) and high levels of repressive mark H3K9me2 along with DNA hypermethylation in the promoter. In *Rhox5 *low expresser cells, we typically observed modest levels of both active and repressive histone marks along with moderate DNA methylation. In *Rhox5 *highly expressed CT26 cancer cells, we observed DNA hypomethylation along with high levels of both active and repressive histone marks. Epigenetic drugs (retinoic acid and MS-275) induced F9 cell differentiation with enhanced *Rhox5 *expression and dynamic changes of epigenetic marks. Finally, *Rhox5 *knockdown by small hairpin RNA (shRNA) in CT26 colon cancer decreased cell proliferation and migration *in vitro *and tumor growth *in vivo *.

**Conclusions:**

Both DNA methylation and histone methylation/acetylation play key roles in modulating *Rhox5 *expression in various cell types. The stem cell-like "bivalent domain", an epigenetic feature originally identified in key differentiation genes within stem cells, exists in the *Rhox5 *gene promoter in not only embryonic stem cells but also cancer cells, cancer stem cells, and differentiated Sertoli cells. As *Ras *signaling-dependent *Rhox5 *expression promotes tumor growth, *Rhox5 *may be an ideal target for therapeutic intervention in cancer.

## Background

The reproductive homeobox on X-chromosome (*Rhox *) gene cluster in mouse contains 33 known genes [1], and three members of this gene family (*Rhox2, Rhox4b and Rhox5 *) are crucial for self-renewal and differentiation of embryonic stem (ES) cells [2-4]. The founding member of *Rhox *gene cluster, *Rhox5 (formerly pem)*, is expressed in early embryos and ES cells [5-7], embryonic carcinoma (EC) cells, and primordial and pre-muscle stem cells [8]. Intriguingly, *Rhox5 *is predominantly expressed in female blastocysts from the paternally inherited X chromosome [7], yet the paternal copy is silent in placenta cells [9]. In adult mice, *Rhox5 *expression is restricted to germline tissues in both male and female and is silenced in most somatic tissues [1,10,11]. *Rhox5 *is expressed from its two promoters, a distal promoter (Pd) and a proximal promoter (Pp), that give rise to transcripts with different 5'-ends encoding the same protein. The transcription from Pp depends on both androgen receptor and androgen [10]. *Rhox5 *plays an essential role in self-renewal and differentiation of ES cells. It has been shown that *Rhox5 *over-expression is able to maintain murine ES cells in a pluripotent state in a leukemia inhibitory factor-independent manner [6], and can also block ES cell differentiation [3,11]. It promotes differentiation and survival of germ cells in germline tissues [10]. Targeted disruption of *Rhox5 *increases male germ cell apoptosis and reduces sperm production, sperm motility, and fertility [12].

*Rhox5 *is expressed not only in established cancer cell lines [13-15], but also in cancers *in vivo*, e.g., adenomas and carcinomas in the APC^Min/+ ^mice and large intestine tumors of Msh2-deficient mice conditionally expressing K-ras (V12) [16,17]. The Pd promoter was regarded as the promoter directing the aberrant expression in tumor cells [10].

Rhox5 may exert important functions in cancer based on the following evidence. First, partners for Rhox5 include: menin, a tumor suppressor [18], prosaposin, a multifunctional protein [19], and the cell division cycle 37 (Cdc37) homolog protein [20]. Second, Rhox5 also mediates transcriptional repression of the netrin-1 receptor gene *Unc5c*, a tumor suppressor in colorectal cancer [21]. Third, *Rhox5 *gene Pd activity in tumor cells requires Ras signaling [22]. Fourth, in a colon adenoma model induced by conditional activation of K-ras^V12 ^in *Msh2 *knockout mice, *Rhox5 *is one of three genes significantly up-regulated [17]. Finally, Rhox5 renders tumor cells resistant to apoptotic cell death induced by anticancer therapies [23]. In addition, it may play a role in cancer initiating cells (or cancer stem [CS] cells) [24]. CS cells are cancer cells that possess characteristics associated with normal stem cells. They have the ability to give rise to all cell types found in a particular tumor. It is possible that ES and CS cells share some key regulatory genes that are tightly regulated by similar epigenetic mechanisms.

While there are a total of 33 known *Rhox *genes clustered in the X chromosome in mouse [1], only two *RHOX *genes have been characterized in humans: *RHOXF1 *(originally called OTEX and hPEPP1) and *RHOXF2A *(originally hPEPP2) [25,26]. While there is no human homolog of mouse *Rhox5*, human *RHOXF1 *is closest to murine *Rhox5 *in terms of chromosomal location of the gene, tissue expression profiles, and potential functions. *RHOXF1 *is expressed at relatively high levels in human ES cells and adult germline stem cells [27]. It is expressed in human colorectal cancer and testicular seminoma *in vivo *[28,29], as well as in some cancer cell lines [15,26]. Therefore, it is possible that *Rhox5 *and *RhoxF1 *may have comparable functions despite low sequence conservation and therefore they may be considered orthologues.

DNA methylation regulates gene expression in normal mammalian development [30,31]. In cancer, aberrant promoter hypermethylation plays a major role in transcriptional silencing of critical growth regulators such as tumor suppressor genes [32,33], while aberrant promoter hypomethylation upregulates germline genes (such as *Rhox5 *) that are normally expressed in embryo stages and stem cells yet silenced in all or most somatic tissues [34,35]. Histone modifications together with DNA methylation in the chromatin regulate many regulatory genes [36,37]. All known acetylations of histones are correlated with transcriptional activation [38]. Histone methylations at lysine and arginine residues are another class of epigenetic marks [39,40]. A recent high-resolution profiling study in the human genome indicated that H3K4 trimethylation and the monomethylations of H3K9, H3K27, H3K79, H4K20 and H2BK5 are linked to gene activation, whereas trimethylations of H3K27, H3K9 and H3K79 are linked to repression [40]. In addition, a "bivalent domain" (repressive mark H3K27me3 and permissive mark H3K4me2/me3) marks key developmental genes in ES cells [41,42]. This chromatin bivalent domain in stem/progenitor cells predisposes tumor suppressor genes to DNA hypermethylation and heritable silencing [43-45].

*RHOX5 *may be regulated by epigenetic mechanisms. First, DNA methylation regulates long-range silencing of *Rhox *gene cluster including *Rhox5 *during the post-implantation development of mice [46]. Second, *Rhox5 *could be upregulated in ES cells and embryonic fibroblast cells by inactivation of DNA methyltransferase genes [46,47], or in ES cells null for linker histone H1 [48]. While this paper was under revision, Wilkinson, MacLean, and coworkers showed that the *Rhox *gene cluster is imprinted and regulated by histone H1 and DNA methylation in ES cells [9]. Third, *Rhox5 *is one of the X-linked cancer-germline (CG) genes, many of which are regulated by DNA methylation [14,35,49]. Finally, we have demonstrated that epigenetic drugs could upregulate *Rhox5 *in cancer cells through enrichment of active histone marks in the promoter region preferentially with DNA demethylation [15].

We and our collaborators have previously investigated epigenetic regulation of genes in normal development and cancer [15,35,50-52]. In this study, we have confirmed that *Rhox5 *is expressed in ES cells, EC cells, and cancer cells. We found that *Rhox5 *is expressed in side population (SP) cells that enrich for cancer stem/progenitor cells. We have examined the epigenetic marks in the promoter region, including both DNA methylation and histone acetylation (H3ac, H4ac and H3K9ac) and methylation (H3K4me2, H3K9me2 and H3K27me3), and related them to levels of expression in various cells types. We showed that epigenetic drugs could induce differentiation of F9 teratocarcinoma cells, but not SP cells, with *Rhox5 *upregulation and concurrent epigenetic changes. Finally, we demonstrated that *Rhox5 *gene knockdown by small hairpin RNA (shRNA) in CT26 colon cancer cells resulted in reduced tumor cell migration and cell proliferation *in vitro *and attenuated tumor growth *in vivo *.

## Results

### Expression of *Rhox5 *gene in ES cells, somatic cells and cancer cells

**Figure 1:**
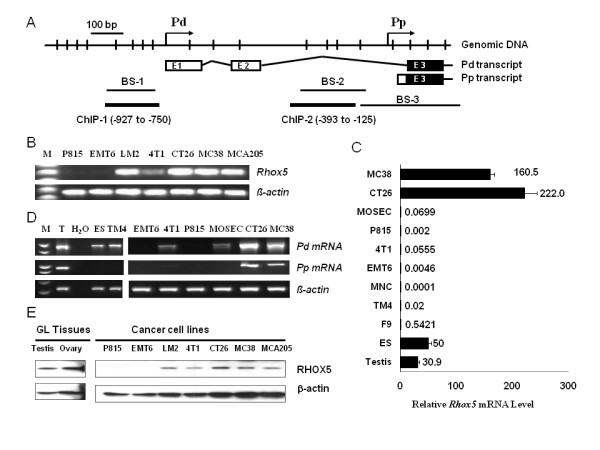
***Rhox5 *expression in ES cells, somatic cells and cancer cells**. (A). Schematic presentation of the Pd and Pp promoter regions of the *Rhox5 *gene. Vertical bars represent CpG dinucletides and open and filled boxes indicated non-coding and coding exons. Three segments (BS-1, BS-2, BS-3) covering CpG dinucleotides subject to bisulfite sequencing and corresponding regions for ChIP assay (ChIP-1 and ChIP-2) were also indicated. (B). *Rhox5 *mRNA in representative cancer cell lines was detected by semi-quantitative RT-PCR. M: PCR marker. (C). Relative *Rhox5 *mRNA levels as normalized to 1.0E4 copies of *GAPDH *mRNA. Real-time RT-PCR was performed in triplicates and data from multiple experiments are presented as mean +/- s.d. The data for ES cells were obtained from single RT-PCR in triplicates. (D). Transcripts from either Pd or Pp were determined by promoter-specific RT-PCR. Lane M: DNA MW Markers; T: testis; H_2 _O: water instead of RNA was used in RT. (E). Western blot analysis of Rhox5 in germline tissues and representative cancer cell lines.

*Rhox5 *gene transcription is controlled by dual promoters, Pd and Pp, producing mRNAs with different 5' ends yet encoding the same protein (Figure 1A). We initially examined *Rhox5 *expression in cancer cells as well as in ES cells and germline tissues. As shown in Table 1, *Rhox5 *mRNA was detected in all 26 cancer cell lines tested. These cancer lines were derived from 12 different tissues. Two cancer cell lines (EMT6 and P815) generated faint bands after 35 cycles of PCR following reverse transcription (RT) (Figure 1B). In contrast, another cancer-germline gene, P1A, which we studied previously, was expressed in a much smaller fraction of cancer cell lines. We then quantified *Rhox5 *mRNA from representative tissues or cells by RT-qPCR (Figure 1C). Testis tissue expressing *Rhox5 *mRNA was utilized as a positive control. ES and F9 EC cells expressed low levels of *Rhox5 *mRNA. Normal somatic cells such as mononucleocytes (MNC) did not express *Rhox5 *mRNA. *Rhox5 *expression in cancer cells varied over a wide range, with high levels in CT26 and MC38 cells and extremely low levels in EMT6 and P815 cells.

**Table 1:** *Rhox5 *and *P1A *mRNA expression in mouse cancer cells

Tumor type	Cell line	*Rhox5 *	*P1A *	Tumor type	Cell line	*Rhox5 *	*P1A *
**Mammary**	4T1	+	-	**Lung**	LLC	+	-

	C3-L5	+	-		LM2	+	-

	C127I	+	-		M109	+	-

	EMT6	+/-	-	**Fibrosarcoma**	MCA102	+	-

	MM2MT	+	-		MCA205	+	-

	TS/A	+	-	**Hepatoma**	Hepa1-6	+	-/+

	TUBO	+	-	**Lymphoma**	A20	+	+

**Colorectal**	CA07/A	+	-		EL4	+	+

	CA51	+	+	**Mastocytoma**	P815	-/+	+

	CMT93	+	-	**Melanoma**	B16	+	-

	CT26	+	-	**Ovarian**	MOSEC	+	-

	MC38	+	-	**Pancreatic**	Panc02	+	-

**Leukemia**	L1210	+	-	**EC**	F9	+	+

* Footnote: "+" represents a strong while "-/+" very weak and "-" undetectable signals of cDNA amplification after 35 cycles of PCR following reverse transcription (RT). The status of *P1A *expression in some cancer cell lines has been reported in our previous study [35].

We next analyzed promoter-specific transcription from both Pd and Pp of *Rhox5 *gene in selected normal cells and cancer cells by promoter-specific RT-PCR as described previously [15]. As shown in Figure 1D, testis tissue utilized both Pd and Pp for transcription, while ES cells utilized the Pd promoter for transcription. TM4 Sertoli cells utilized mainly Pd, consistent with results from a previous study [53]. Among the selected group of cancer cells, CT26, MC38, and 4T1 cells utilized both Pd and Pp for transcription. *Rhox5 *mRNA was barely detectable in EMT6 and P815 cells.

We further confirmed gene expression at the protein level by Western blot analysis (Figure 1E). Both germline tissues (testis and ovary) and selected cancer cells expressed Rhox5 protein (~30 kDa). In contrast, Rhox5 protein was below the level of detection in EMT6 and P815 cancer cells. These results were consistent with those obtained by RT-PCR.

### *RHOXF1 *expression in human primary colorectal cancers

**Figure 2:**
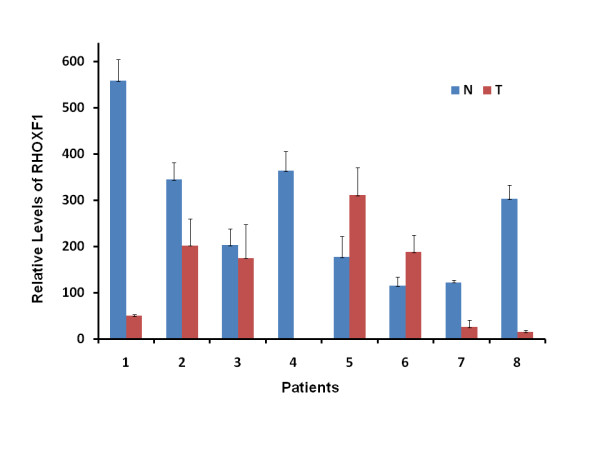
***RhoxF1 *mRNA expression in liver lesions of colorectal cancer and matched liver tissues from colorectal cancer patients**. Total RNA from matched liver tissues (N) and metastatic colorectal cancer (T) was purified and *RHOXF1 *mRNA was quantified by RT-qPCR. Relative *Rhox5 *mRNA levels were expressed as normalized to 1.0E6 copies of β-*actin *mRNA. Bars represent means with s.d. In patient 4, *RHOXF1 *mRNA from tumor (T) was below the level of detection.

We wished to confirm if *RHOXF1 *is expressed in human colorectal cancers, as reported by gene expression profiling [28]. We collected eight matched sets of specimens from patients with metastatic colorectal cancer. These tissues represented liver metastasis and matched normal liver tissues from eight patients. Total RNA was purified from these tissues, and the amounts of *RHOXF1 *mRNA were quantified by RT-qPCR (Figure 2). *RHOXF1 *mRNA was expressed in the normal liver tissues (N), ranging from 122 to 558 copies relative to 1.0E6 copies of β-actin mRNA. In the tumor tissues (T), *RHOXF1 *mRNA was also expressed in 7 out of 8 patients, ranging from 15 to 310 copies of mRNA.

### Correlation of *Rhox *5 gene expression to the histone epigenetic marks in the promoter region of the gene

**Figure 3:**
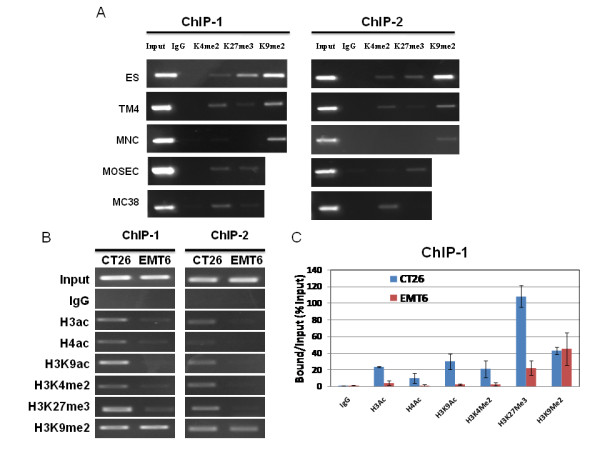
***Rhox5 *is bivalent marked in ES cells, somatic cells and cancer cells**. (A). Histone methylation marks (H3K4me2; H3K27me3 and H3K9me2) on *Rhox5 *promoter regions were determined by ChIP assays. Locations of ChIP-1 and ChIP-2 regions were illustrated in the cartoon of Fig. 1A. (B). Histone acetylation and methylation marks on promoter regions in cancer cells with *Rhox5 *expression at high (CT26) and low (EMT6) levels. (C). Quantification of the data from CT26 and EMT6 cells. Bars are mean with s.d.

We sought to find a correlation between *Rhox5 *gene expression and its epigenetic marks in the promoter region (Table 2). Initially we examined histone modifications in ES and other cells by ChIP assays. In ES cells, there was a low level of H3K4me2 and higher levels of H3K27me3 and H3K9me2 marks on ChIP-1 region (Figure 3A). In Pd region (ChIP-2 region), the pattern was similar. This pattern of histone marks would correlate with the low level of expression seen in ES cells. In gene-silenced MNC and mammary epithelial cells, as well as P815 cancer cells with extremely low level of *Rhox5 *mRNA, they revealed high levels of H3K9me2 together with low levels of H3K27me3. The active marks were either undetectable or barely detectable (Figure 3A and Additional File 1). Interestingly, we did not detect H3K4me2, H3K27me3, and H3K9me2 marks on *Rhox5 *Pd region in 4T1 cells, although a low level of mRNA was transcribed from Pd in these cells (Additional File 1).

**Table 2:** Summary of locations of key data from various cells

Primary Cells or Cell Lines	RT-PCR	Bisulfite Sequencing	ChIP Assay
ES Cells	Fig. 1	Fig. 4	Fig. 3

Sertoli cells (TM4)	Fig. 1	Fig. 4	Fig. 3

Mononucleocytes (MNC)	Fig. 1	Fig. 4	Fig. 3

CT26 (colon cancer)	Fig. 1	Fig. 4	Fig. 3

MC38 (colon cancer)	Fig. 1	Fig. 4	Fig. 3

4T1 (mammary cancer)	Fig. 1	Fig. 4	AF. 1

EMT6 (mammary cancer)	Fig. 1	Fig. 4	Fig. 3; Fig. 5

P815 cells (mastocytoma)	Fig. 4	AF. 2	Fig. 5; AF. 1

EMT6 & P815 cells treated with epigenetic drugs	Fig. 4	n.d.	Fig. 5

F9 (embryonic carcinoma)	Fig. 1; Fig. 5	n.d.	Fig. 5

MOSEC (ovarian cancer)	Fig. 1; Fig. 6	Fig. 4	Fig. 3

MOSEC SP	Fig. 6	n.d.	Fig. 6

n.d., not done. AF: Additional File.

We chose to compare the histone marks in two cancer cell lines with either the highest (CT26) or the lowest (EMT6) *Rhox5 *expression (Figure 3B & C). The active marks (H3ac, H4ac, H3K9ac and H3K4me2) are high in CT26 cells, and very low in EMT6 cells. Interestingly, we detected relative high levels of repressive marks (H3K9me2 and H3K27me3) in both CT26 and EMT6 cells.

We have also paid attention to the "bivalent domain" chromatin structure in the promoter region. The H3 K4me2 and K27me3 bivalent marks exist not only in undifferentiated ES cells, but also in germline-tissue derived somatic cells (TM4) and some cancer cells (MOSEC, CT26 and MC38).

### Strong correlation of promoter DNA methylation with *Rhox *5 gene expression

**Figure 4:**
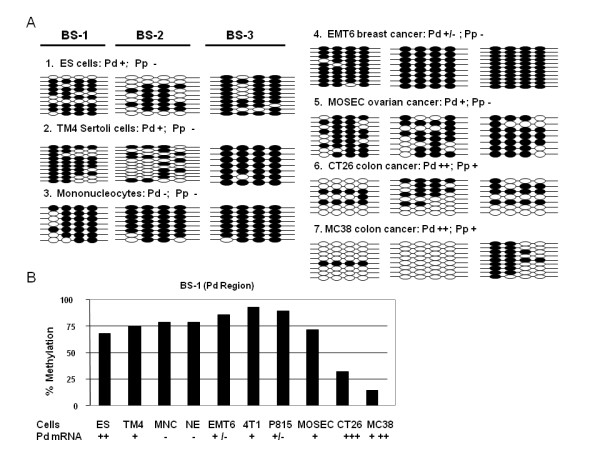
**Promoter DNA methylation pattern and *Rhox5 *mRNA expression level**. **(**A). DNA methylation status in Pd, Pp, and TSS regions in different cell types including ES, somatic cells, and cancer cells. Each column represented one CpG site and one row indicated separate clones picked for sequencing. Open and filled circles indicate individual unmethylated and methylated CpGs, respectively. (B). Correlation of Pd DNA methylation status versus gene transcription in 10 different cell types. Cell types are, NE: normal mammary epithelial cells MM3MG. Six cancer cell lines used are, EMT6, 4T1, P815, MOSEC, CT26 and MC38. -: non-detectable; +/-: extremely low; +: low; ++: moderate; +++: high.

We wished to determine DNA methylation status in the promoters of *Rhox5 *gene in the same set of cell types. Both Pd and Pp promoters of the gene are CpG-poor and contain no CpG islands (GenBank accession: AF410462) [54]. Specific primers were selected to amplify bisulfite-treated genomic DNA from ten lines of cells including ES cells, somatic cells, and cancer cells. These primers covered DNA segments in the Pd, Pp, and translation start site (TSS) regions (BS-1, BS-2 and BS-3 regions, respectively), covering four CpG dinucleotides each (see Figure 1A). As shown in Figure 4 (A & B), both ChIP-1 (Pd) and TSS regions were relatively hypermethylated in ES cells. As *Rhox5 *is expressed at a low level from Pd in ES cells, our results suggested that DNA hypermethylation and a moderately repressive pattern of histone epigenetic marks together dictated a low level of *Rhox5 *expression. TM4 and MOSEC cells had similar epigenetic patterns as ES cells, and this also correlated with low level of *Rhox5 *expression. For CT26 and MC38 cells that express high levels of *Rhox5 *gene, hypomethylated DNA was found in the promoter regions. Data from additional normal and cancer cells were presented in Additional File 2. The percentage of CpG methylation in the Pd region correlated quite well with the levels of Pd mRNA expression in the cells (Figure 4B).

### Differentiation of F9 EC cells induced by epigenetic agents resulted in significant changes of histone marks

**Figure 5:**
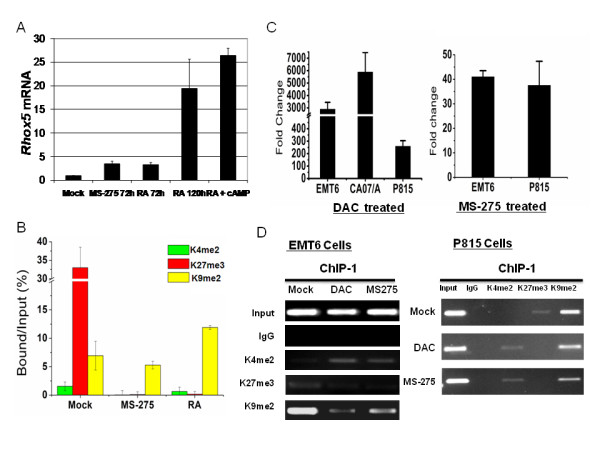
**Upregulation of *Rhox5 *mRNA was associated with dynamic changes of H3K4 and H3K27 methylation marks in Pd region**. (A). Upregulation of *Rhox5 *in differentiating F9 cells as induced by MS-275, RA or RA plus cAMP. The mRNA was quantified by RT-qPCR, and the level of *Rhox5 *mRNA in mock-treated was arbitrarily set as 1.0. (B). Occupancy of H3 K4me2, K27me3, and K9me2 marks in Pd region in mock, MS-275, and RA-treated F9 cells as determined by real-time RT-PCR. (C). Upregulation of *Rhox5 *mRNA in DAC or MS-275-treated cancer cells. Fold change of *Rhox5 *mRNA (as normalized by *Gapdh *) was compared to mock-treated cells. (D). ChIP analysis of K4me2, K27me3 and K9me2 marks at *Rhox5 *Pd region (ChIP-1) in mock and epigenetic drug-treated EMT6 and P815 cancer cells.

A distinct characteristic of genes marked by a bivalent domain is that these genes can change expression levels rapidly during ES differentiation as bivalent marks are resolved to monovalent marks (H3K4me2 or H3K27me3 only), remain bivalent, or disappear altogether [41,42,55]. As a result we sought to study changing patterns of histone epigenetic marks during EC differentiation. The F9 EC cells can be induced to differentiate with upregulation of *Rhox5 *mRNA by retinoic acid (RA), RA plus cAMP, or valproic acid. All these agents exhibit properties of epigenetic modulators [8,56,57]. The HDAC inhibitor MS-275 can induce p21-dependent growth arrest and differentiation of human leukemia cells at lower doses [58]. We demonstrated that both MS-275 and RA treatment induced *Rhox5 *mRNA 3-fold by 72 h, and RA plus cAMP could induce *Rhox5 *20-25-fold in 5 days (Figure 5A). These differentiated cells displayed dramatically reduced tumorigenicity in nude mice (Additional File 3). In undifferentiated F9 EC cells, the Pd promoter was marked with low levels of K4me2, but higher levels of K27me3 and K9me2 (Figure 5B). Upon induced differentiation by either drug, K27me3 disappeared and K4me2 was reduced (p < 0.05), while K9me2 was not significantly affected.

### *Rhox5 *induction in silenced cancer cells by epigenetic drugs via increased permissive and decreased repressive marks

We sought to study the dynamic changes of histone marks along with *Rhox5 *gene induction in cancer cells treated with DAC or MS-275. CA07/A, EMT6 and P815 cancer cells express very low levels of *Rhox5 *mRNA (Figure 1 and Table 1). Upon treatment with decitabine (DAC) or MS-275, *Rhox5 *mRNA was significantly upregulated, ranging from 40 to 3000-fold (Figure 5C). We then analyzed the histone marks in the Pd in cancer cells without or with drug treatment (Figure 5D). In mock-treated EMT6 and P815 cancer cells, there were elevated levels of H3K9me2, very low levels of H3K27me3, and undetectable levels of H3K4me2. After drug treatment, significant induction in H3K4me2 and reduction in H3K9me2 was observed, yet H3K27me3 remained low or reduced.

### *Rhox5 *was expressed in SP and NSP of cancer cells with bivalent histone marks

**Figure 6:**
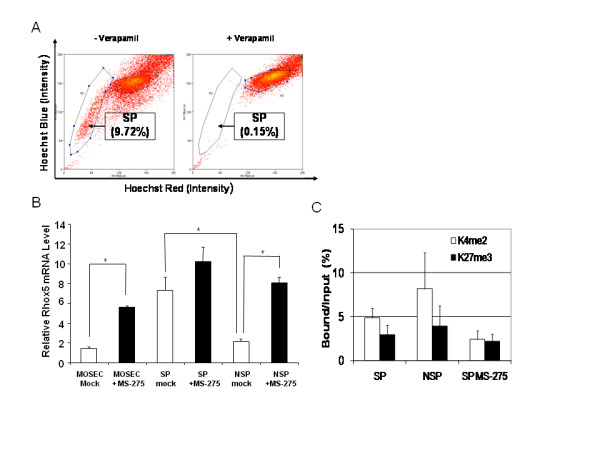
***Rhox5 *mRNA expression and K4 and K27 bivalent marks in mock or MS-275 treated SP and NSP cells**. (A). Existence of SP and NSP in MOSEC cancer cells. (B). Regulation of *Rhox5 *mRNA expression in mock or MS-275-treated MOSEC, SP, and NSP cells. *: P < 0.05. (C). Occupancy of K4 and K27 marks on the chromatin of Pd ChIP-1 region in SP, NSP, and MS-275-treated SP cells. Experiments were performed in triplicate; error bars depict SEM.

We next examined whether *Rhox5 *was expressed in cancer stem (CS)/progenitor cells and whether there was an associated bivalent chromatin pattern. The SP from primary cancers and cancer cell lines has been shown to be enriched for CS/progenitor cells [59]. Hoechst 33342 dye exclusion was performed with verapamil as a specific inhibitor of H33342 transport in order to identify SP. We initially chose CT26 colorectal cancer cells and showed that there was a small fraction of SP (~1%) and that *Rhox5 *was expressed in both SP and NSP (data not shown). Due to the number of SP cells needed to properly perform the ChIP assays, it was difficult to obtain sufficient SP cells from this colorectal cancer cell line. Thus we utilized ovarian cancer cells because ovarian cancer cells contain a relatively large SP that is enriched for CS/progenitor cells [60]. Indeed we showed that the MOSEC ovarian cancer cell line contained 9.7% of SP and that this population could be blocked by verapamil (Figure 6A). RT-qPCR demonstrated that SP expressed *Rhox5 *mRNA about 3-fold higher than NSP from MOSEC cancer cells (Figure 6B). We examined the possibility of *Rhox5 *upregulation in SP by the epigenetic drug MS-275. There was a 3~4-fold induction of *Rhox5 *mRNA in both the original MOSEC and NSP cells by MS-275. However, there was no significant up-regulation of *Rhox5 *in MS-275-treated SP cells (Figure 6B). We also examined two key histone marks (K4me2 and K27me3, the bivalent marks) and found that the Pd promoter was marked by both K4me2 and K27me3 in both SP and NSP from MOSEC cells. As expected, MS-275 treatment did little to change the pattern of these two histone epigenetic marks in SP cells (Figure 6C).

### *Rhox5 *knockdown attenuated cell proliferation and cell migration in vitro and tumor growth in vivo

Little is known concerning *Rhox5 *function in cancer cells. Therefore we wished to explore the functions of *Rhox5 *in cancer cells. We selected a colon cancer model (CT26) for *Rhox5 *functional analyses since our initial results indicated that CT26 cells express a high level of *Rhox5 *mRNA.

**Figure 7:**
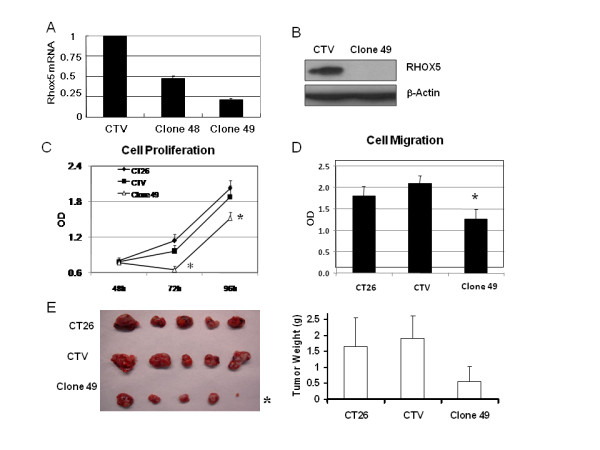
**ShRNA-mediated knockdown of *Rhox5 *reduced cell migration and attenuated growth of CT26 cancer cells**. (A). Efficiency of *Rhox5 *knockdown of shRNA clones with different target sequences against *Rhox5*. Lentiviral particles with control empty vector (CTV) or 2 different target sets (clone 48, 49) of shRNA were used to infect CT26 cells. After 3^rd ^round of puromycin selection, total RNA was purified and analyzed with RT-qPCR and expression of relative *Rhox5 *mRNA was compared to the empty vector control (set as 1.0). (B). Reduced Rhox5 protein level in *Rhox5 *shRNA-treated clone 49 cells. Representative result of Western blot film exposed for a short time (10 seconds) was shown. Rhox5 protein in clone 49 was detected in a longer exposure. (C). Proliferation of CT26 cells stably-transfected with control or targeted shRNA at different time points *in vitro*. (D). Migration ability of CT26 cells stably-transfected with control or targeted shRNA. (E). Tumor volumes (left side) and tumor weight (right side) of control and targeted shRNA stably-transfected CT26 cells at day 19 after inoculation in athymic nude mice. *: p < 0.05 when compared with control groups.

We used lentivirus-mediated shRNA against *Rhox5 *to knockdown the expression of this gene. As shown in Figure 7A, shRNA clone 49 demonstrated a higher knockdown efficiency than clone 48 (80% versus 50%) as determined by RT-qPCR. Western blot analysis confirmed that Rhox5 protein was greatly reduced in clone 49 (Figure 7B). We chose clone 49 for further characterization *in vitro *and *in vivo *. Cell proliferation was significantly decreased at 72 and 96 h following knockdown compared to the parental CT26 cells and corresponding control lentiviral vector transduced (CTV) CT26 cells (p < 0.05) (Figure 7C). Cell migration ability in clone 49 cells was also significantly reduced (p < 0.05; compared to CT26 and CTV, respectively) (Figure 7D). We further examined the property of tumor growth from shRNA knockdown and parental CT26 cells in a subcutaneous tumor model in athymic nude mice. Tumor growth was slower over time in mice inoculated with clone 49 compared to those with parental CT26 cancer cells or CTV CT26 cells. At the time of sacrifice (day 19), both tumor volumes and tumor weights were significantly reduced in the clone 49 group compared to the two control groups (p < 0.05) (Figure 7E).

## Discussion

The *Rhox *gene cluster is essential for development, and three members (*Rhox2, Rhox4 and Rhox5 *) have important functions for pluripotency of ES cells. In a recent study, it has been demonstrated that *Rhox2 *and *Rhox4 *genes, both expressed at low levels in ES cells, are marked by neither K4 nor K27 trimethylation of histone H3 in ES cells [61]. This suggests that DNA methylation is one of the major repressive mechanisms for those genes that lack both H3 K4/K27 trimethylations. Previous studies suggest that DNA methylation is involved in *Rhox5 *gene regulation, yet histone modifications around the promoter region of the gene in correlation to gene expression have not been examined.

In this study, we undertook the task of analyzing the epigenetic marks in the *Rhox5 *gene promoter region, and we related these modifications to *Rhox5 *expression levels in ES cells, germline-tissue-derived Sertoli cells, cancer cells, and cancer stem/progenitor cells, as well as *Rhox5 *-silenced somatic cells. We had three main goals in mind. First, we wanted to examine both DNA methylation patterns and histone marks around the promoter region to determine if the epigenetic patterns would correlate with *Rhox5 *expression in those cells. Second, we wish to examine whether the "bivalent domain" epigenetic feature originally identified in key developmental genes in ES cells also existed in the *Rhox5 *gene in both ES cells and other types of cells such as cancer stem cells. Finally, since *Rhox5 *is expressed in most, if not all, of the cancer cell lines and in colorectal cancer *in vivo*, it was of great interest to begin to uncover its potential function in cancer.

The general conclusion from our current study is that the sum of both active and repressive epigenetic marks together dictates the levels of *Rhox5 *mRNA expression in a particular cell type or cell line. DNA hypermethylation together with repressive histone modifications dictate the silencing or extreme reduction in *Rhox5 *expression in normal mononucleocytes (MNC) or EMT6 cancer cells. In cells expressing low levels of *Rhox5 *such as ES cells, F9 cells, and TM4 cells, DNA is moderately methylated, and the histone epigenetic marks profile shifted to a more neutral state. These cells displayed both active marks and repressive marks, even though the exact marks and levels of these marks varied from one cell type to another. The existence of a "bivalent domain" represents such an epigenetic feature in these cells. In cells (CT26 and MC38) with high levels of *Rhox5 *expression, DNA is hypomethylated, and the active histone marks are also elevated, consistent with high levels of *Rhox5 *mRNA. Surprisingly, we also detected high levels of repressive histone marks.

We found the "bivalent domain" chromatin epigenetic structure in the *Rhox5 *promoter not only in ES cells and SP cells enriched for cancer stem/progenitor cells, but also in cancer cells and fully differentiated germline tissue-derived somatic Sertoli cells. Our study is not the first to show that the bivalent chromatin signature is present in somatic cells. Roh et al. have shown that about 59% of gene promoters studied in primary human T cells contain bivalent marks [62]. In the human foreskin fibroblast BJ cell line, bivalent marks exist in some lineage specific genes [63]. In cancer cells, *SFRP *and *GATA *genes are marked by a bivalent chromatin domain, and the authors defined this as a "stem cell-like chromatin structure" [60]. For *Rhox5*, we also found this stem cell-like chromatin structure in three cancer cell lines (CT26, MC38 and MOSEC). Populations of cancer cells are heterogeneous and contain only a small number of cancer stem cells that possess the capacity to maintain self-renewal and undifferentiated status. We further sorted two cell populations (SP and NSP) from MOSEC cells. Surprisingly, both fractions of cells contain the bivalent domain in the *Rhox5 *gene promoter.

One of our initial aims was to induce differentiation of CS/progenitor cells by HDAC inhibitors [64], in order to examine *Rhox5 *gene expression during differentiation and to explore this as a potential therapeutic approach. F9 EC cells are considered by many to be the malignant stem cells of teratocarcinoma [65]. We have confirmed that F9 cells can be differentiated into "normal cells" by epigenetic drugs such as RA and MS-275. Upon such an induction of differentiation these cells display a benign phenotype as the tumor formation in nude mice was retarded. The *Rhox5 *gene was upregulated and the bivalent marks disappeared or were greatly reduced. This is consistent with findings by other investigators that a fairly large group of active genes contain neither of the two histone marks [40,66]. The remodeling of these histone marks in the promoter may be related to the differentiation status and/or particular cell type after induction of differentiation.

When MS-275 was applied to the CS/progenitor-enriched SP cells from MOSEC ovarian cancer, it failed to up-regulate *Rhox5 *and did not reduce the bivalent chromatin pattern in the gene. In this and other studies, SP cells were isolated based on the property of high levels of ABCG2 pump molecule capable of mediating the active efflux of numerous anticancer drugs and the dye Hoechst [59]. These SP cells could mediate the efflux of MS-275 similar to what occurs with other drugs. This might explain why SP cells failed to respond to MS-275-induced cell differentiation.

We showed that *Rhox5 *knockdown by shRNA in CT26 colon cancer decreased cell migration and cell proliferation *in vitro *and tumor growth *in vivo *. This is reminiscent of the previous results that targeted disruption of *Rhox5 *increased male germ cell apoptosis and reduced sperm production, sperm motility, and fertility [12]. What are the downstream molecules and how does *Rhox5 *knockdown affect downstream signaling in cancer? One gene directly targeted by *Rhox5 *is *Unc5c*, a tumor suppressor frequently silenced by DNA methylation in colon cancer [21,67]. In CT26 colon cancer cells, *Unc5c *is not expressed, and *Rhox5 *knockdown by shRNA did not change *Unc5c *expression (data not shown). Instead, the attenuated CT26 cancer growth and migration by *Rhox5 *knockdown may be mediated by *Ras-ERK *signaling pathway. Evidence for this could be found in a colon adenoma model induced by conditional activation of K-ras^V12 ^in *Msh2 *knockout mice in which *Rhox5 *is one of three genes significantly upregulated [17]. Interestingly, P1A, another epigenetically regulated and X-linked cancer-germline gene we have studied previously [35], was also upregulated in this K-ras^V12 ^/Cre//*Msh2 *^- ^tumor model. A recent study showed that ectopic expression of *Rhox5 *in cancer cells induced a significantly increased extracellular signal-regulated kinase (ERK) activity and multiple resistance to various apoptotic pressures [23]. In addition, it has been shown that Ras signaling activates *Rhox5 *transcription through its Pd promoter [68]. Oncogenic Ras signaling also induces tumor promoting genes and directs epigenetic inactivation of tumor suppressor genes [69,70]. Another downstream component of the Ras signaling pathway, NF-κB, promotes breast cancer cell migration and thus metastasis by inducing chemokine receptor CXCR4 [71]. Therefore, our finding that *Rhox5 *knockdown attenuated tumor cell growth and cell migration fits a hypothetic Ras tumor promoting signaling pathway in which ERK1/2, NF-κB, and Rhox5 function downstream. Further studies will shed more light on Rhox5 function in precancerous lesions and in cancer progression of colon malignancy. In addition, *Rhox5 *is widely expressed in cancer cells and cancer stem/progenitor cells, and can be selectively induced or suppressed by epigenetic agents. Thus, *Rhox5 *could serve as an ideal target for therapeutic interventions including shRNA therapy, cancer immunotherapy, and epigenetic therapy.

The closely related human gene *RHOXF1 *has been shown to be expressed in ES cells and adult germline stem cells, some established cancer lines and in primary metastatic colorectal cancer. Its expression pattern is consistent with potential roles in ES cells, adult tissue stem cells, and possibly cancer stem cells, despite the fact that we know little, if any, of its biological functions. Efforts to elucidate the functions of *RHOXF1 *in the biology of cancer and reproduction and to explore RHOXF1 as a potential therapeutic target should be undertaken.

## Methods

### Cell culture and human tissues

Many cancer cell lines have been used in our previous studies [15,35]. The F9 EC cells were obtained from the American Type Culture Collection (Manassas, VA). In order to maintain F9 undifferentiated status, F9 cells were grown on gelatin-coated tissue culture plates. All cells were cultured in the recommended culture media supplemented with 5% or 10% fetal bovine serum (FBS), plus penicillin and streptomycin.

Undifferentiated mouse ES cells (genotype 129*129, passage 18) were purchased from Open Biosystems (Huntsville, AL). They were used directly for analysis of gene expression, bisulfite sequencing, and ChIP assays.

The specimens of human colorectal cancer and matched normal tissues were collected under the UPCI protocol # 02-077, with consent of the patients.

### Flow cytometry

To identify and isolate the side population and non-side population cell fractions, cancer cells were harvested, washed, and suspended at 1.0E6 cells/ml in Hanks balanced salt solution (HBSS) (Invitrogen, Carlsbad, CA) as described [72]. The cells were labeled with Hoechst 33342 (Invitrogen) at a concentration of 5.0 μg/ml in the absence and presence of 50 μM verapamil (Sigma, St. Louis, MO). The labeled cells were incubated for 90 min at 37°C. After washing with HBSS once, the cells were counterstained with 1.0 μg/ml 7-AAD (Becton Dickinson, Franklin Lakes, NJ) to label dead cells. The cells were analyzed by using a MoFlo cell sorter (Beckman Coulter, Fort Collins, CO).

### Drug treatment

*Rhox5 *gene induction was performed by treating cancer cells with 5-aza-2-deoxycytidine (DAC, 2.0 μM for 48 h) or MS-275 (2.0 μM for 72 h) [15]. Cells were plated in 100 mm culture plates to obtain ~20% confluence. After overnight incubation, cells were treated daily with drugs at different concentrations for 48 ~ 72 h. To induce differentiation, F9 cells were cultured in gelatinized plates in the presence of 0.1 μM retinoic acid (RA) (Sigma) or RA plus 1.0 mM cAMP (Sigma) as described [8].

### RNA isolation, RT-PCR and RT-qPCR

Total RNA purification, RT-PCR, and RT-qPCR were performed as described previously [15]. RT-qPCR was performed with an ABI StepOnePlus real-time PCR system (Applied Biosystems, Foster City, CA). The copy numbers of mRNA were determined with relative quantitation by the comparative Ct method using the software with the machine.

### Western blot analysis

Western blot analysis was performed as described [15]. Briefly, protein extract was prepared from tumor cells and from ovary and testis tissues of BALB/c mice. Twenty micrograms of protein was resolved on 12% SDS polyacrylamide gels and transferred to immobilon-P PVDF membrane (Millipore, Billerica, MA). The resulting blots were blocked with 5% nonfat dry milk and probed with antibodies specific for Rhox5 (Abcam, Cambridge, MA) and ß-actin (Sigma).

### Isolation of genomic DNA and bisulfite sequencing

Genomic DNA from cell lines was extracted using a QIAamp DNA mini kit (Qiagen, Volencia, CA). DNA from spleen mononucleocytes (MNC) of a BALB/c mouse was extracted using a DNeasy Tissue kit (Qiagen). Bisulfite modification of DNA, subcloning, and sequencing of converted DNA were performed as described [15].

### Chromatin immunoprecipitation (ChIP) assay and real-time PCR

ChIP assays were performed using EZ-ChIP kits (Millipore, Billerica, MA) [15]. The following ChIP-grade antibodies were used: anti-acetyl histone H3 [H3ac] and anti-acetyl histone H4 [H4ac] (Millipore), anti-acetyl histone H3 lysine 9 [H3K9ac], anti-dimethyl histone H3 lysine 4 [H3K4me2], anti-dimethyl histone H3 lysine 9 [H3K9me2], anti-trimethyl histone H3 lysine 27 [H3K27me3], and an isotype control IgG (All from Abcam). In earlier experiments, histone 3 K4, K27, and K9 methylation in the ChIP-1 region was quantified by semi-quantitative PCR gel density analysis. In all later experiments, real-time PCR was used to quantify the amounts of DNA fragment in the ChIP assays. Specific primer sets were designed to amplify *Rhox5 *gene ChIP-1 (Pd) and ChIP-2 (Pp) regions. Most primer sequences are listed in Additional File 4. For *Rhox5 *Pd real-time PCR, 2.0 μl of DNA was added to PCR reaction systems using a QuantiTect SYBR Green PCR kit (Qiagen). We performed quantitative PCR data analysis of ChIP assay using a formula described in the User Manual of ChampionChIP™ kits (SA Biosciences, Frederick, MD, USA). Briefly, we normalized each ChIP DNA fractions' Ct value to the Input DNA fraction Ct value for the same qPCR Assay (ΔCt) to account for chromatin sample preparation differences. Then we reported ChIP-qPCR results as a "% Input" for characterizing individual experimental samples.

### ShRNA-mediated knockdown of *Rhox5 *gene

Four different lentivirus particles with target shRNA against *Rhox5 *were ordered from Sigma. The best result for knockdown was obtained from clone 49. The shRNA clone 48 sequence is, CCGGAGTGCAGAATTGGTTTAAGATCTCGAGATCTTAAACCAATTCTGCACTTTTTTG. The shRNA clone 49 sequence is, CCGGCAGCGCACTAATTCCTTTGATCTCGAGATCAAAGGAATTAGTGCGCTGTTTTTG). A lentivirus with the corresponding empty plasmid vector (CTV) was used as non-target control. Lentivirus with *Rhox5 *target and non target shRNA was used to infect CT26 cells at MOI of 1.0. After three rounds of puromycin (6.0 μg/ml) selection, stably transduced CT26 cells were selected and *Rhox5 *knockdown was assessed by both real-time RT-PCR and Western blot analysis.

### Cell proliferation and cell migration assays

For cell proliferation assays, 1,000 CT26 cancer cells in 10% FBS-containing DMEM medium were added to each well of a 96-well plate. Cell proliferation was determined by using CellTiter 96 AQueous Non-Radioactive Cell Proliferation Assay Kit (Promega, Madison, WI). The reagent was added directly to culture wells, and following incubation for 4 h at 37°C, absorbance at 490 nm was measured using a 96-well plate reader. For trans-well migration assays, 1 × 10^5 ^serum starved cells in serum-free medium were added to the top chambers of 24-well trans-well plates (Cell Biolabs, San Diego, CA), and growth media containing 10% FBS was added to the bottom chambers. After 12 h of incubation, migrating cells were stained, and absorbance was recorded at 560 nm. Assays were done in triplicates, and the data are presented as the average absorbance of cells.

### *In vivo *tumor growth

Athymic nude mice were ordered from Tacomic Farms, Inc. (Germantown, NY). Mice were housed in standard conditions and given food and water *ad libitum *. The animal study was approved by the Institutional Animal Care and Use Committee of the University of Pittsburgh.

*Rhox5 *and control shRNA lentivirus-stably-transduced CT26 colon cancer cells were injected subcutaneously into hind frank of 5-6 weeks old athymic nude mice (1.0E5 cells per mouse, 5 mice per group). Mice were closely monitored until any one animal possessed a tumor of 2.0 centimeter in diameter. At this time point, tumor volumes of all mice were measured, and mice were sacrificed.

### Statistical analysis

Statistical analysis was calculated using Microsoft Excel or SPSS software. Significance was calculated using Student's *t *-test.

## Competing interests

The authors declare that they have no competing interests.

## Authors' contributions

QL participated in all phases of the project, carried out the majority of the experiments, analyzed the data, and assisted in writing the manuscript. MEO participated in the animal experiments and assisted in editoring the manuscript. DLB participated in the design of this study. ZSG conceived and designed the experiments, and assisted in writing the manuscript. All authors read and approved the final manuscript.

## Supplementary Material

Additional file 1 **The results of ChIP assays with *Rhox5 *promoter regions in MM3MG, P815 and 4T1 cells**. The ChIP assays were performed as those in Figure 2. Shown are one mammary fibloblast (MM3MG) and two cancer cell lines. Click here for file

Additional file 2 **DNA methylation analysis in ChIP-1 and ChIP-2 regions of the gene from MM3MG mammary epithelial cells and P815 cancer cells**. Data of DNA methylation analysis in *Rhox5 *ChIP-1 and ChIP-2 regions are presented. The details are in Figure 3. Click here for file

Additional file 3 **Growth of F9 embryonic carcinoma in nude mice**. Tumor growth in nude mice, inoculated with mock-treated F9 cells (left frank) or MS-275-treated F9 cells (right frank). Click here for file

Additional file 4 **PCR primers used for PCR assays**. Click here for file
