# Supplementary figures and images for: Homeobox gene Rhox5 is regulated by epigenetic mechanisms in cancer and stem cells and promotes cancer growth
Source: Mol Cancer. 2011 May 24;10:63. doi: 10.1186/1476-4598-10-63 (PMC3125390; doi:10.1186/1476-4598-10-63)

## ChIP-1

## ChIP-2

Input IgG K4me2 K27me3 K9me2

Input IgG K4me2 K27me3 K9me2

MM3MG

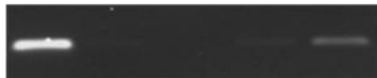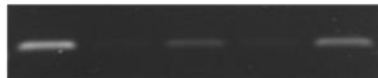

P815

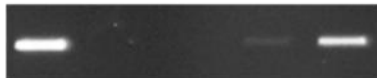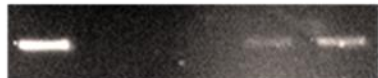

4T1

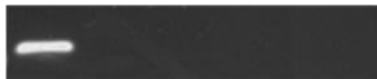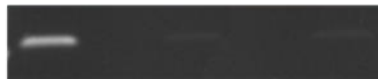

Supplement: Additional file 1 — The results of ChIP assays with Rhox5 promoter regions in MM3MG, P815 and 4T1 cells. The ChIP assays were performed as those in Figure 2. Shown are one mammary fibloblast (MM3MG) and two cancer cell lines. [file 1476-4598-10-63-S1.PDF]

**MM3MG (Mammary epithelial cells): Pd mRNA - ; Pp mRNA -**

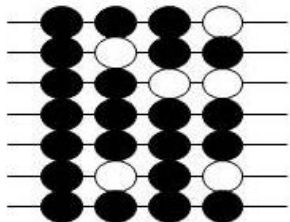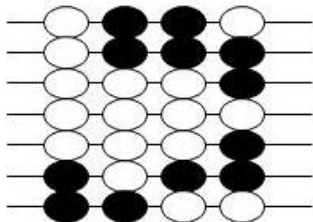

**P815 (Mastocytoma cells): Pd mRNA - ; Pp mRNA -**

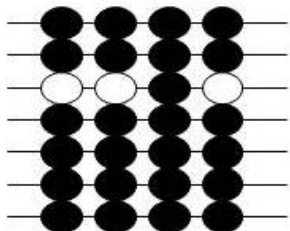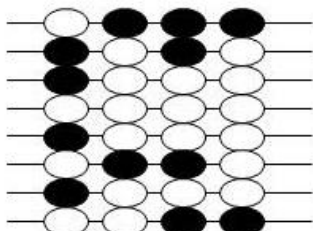

Supplement: Additional file 2 — DNA methylation analysis in ChIP-1 and ChIP-2 regions of the gene from MM3MG mammary epithelial cells and P815 cancer cells. Data of DNA methylation analysis in Rhox5 ChIP-1 and ChIP-2 regions are presented. The details are in Figure 3. [file 1476-4598-10-63-S2.PDF]

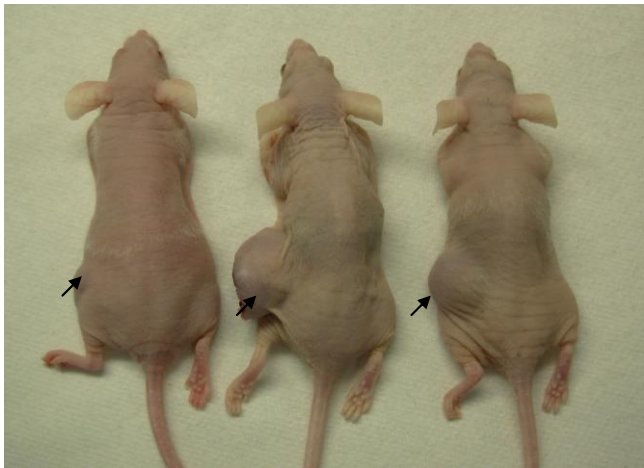

Supplement: Additional file 3 — Growth of F9 embryonic carcinoma in nude mice. Tumor growth in nude mice, inoculated with mock-treated F9 cells (left frank) or MS-275-treated F9 cells (right frank). [file 1476-4598-10-63-S3.PDF]
