# Supplementary material for: Homeobox gene Rhox5 is regulated by epigenetic mechanisms in cancer and stem cells and promotes cancer growth
Source: Mol Cancer. 2011 May 24;10:63. doi: 10.1186/1476-4598-10-63 (PMC3125390; doi:10.1186/1476-4598-10-63)
Supplement: Additional file 4 — PCR primers used for PCR assays. [file 1476-4598-10-63-S4.DOC]

**Additional File 4. PCR primers used for PCR assays**

| **Gene Name** | **Primer Sequences** |
| --- | --- |
| **mRNA expression** | **(for semi-quantitative)** |
| ***Rhox5*-F** | AATGGAAATCCTGGGGGTAG |
| ***Rhox5*-R** | AAATCTCGGTGTCGCAAAAG |
| ***ß-actin*-F** | TGGAATCCTGTGGCATCCATGA |
| ***ß-actin*-R** | TAAAACGCAGCTCAGTAACAGT |
| **Promoter specific mRNA expression** | |
| ***Rhox5*-Pd-F** | TGCACAGTCCTTCAAGCTCACC |
| ***Rhox5*-Pp-F** | AGCTCAGAATCTGCCGAAGC |
| ***Rhox5*-R** | ATCTCACTCCACGACAAGCAGGAC |
| **Bisulfite sequencing** |  |
| ***Rhox5*-Pd-F (BS-1)** | TTTTAGGTGTGAAGAGGTGAGTTAGA |
| ***Rhox5*-Pd-R (BS-1)** | TCACCAAAACAAAAAAAACCATAA |
| ***Rhox5*-Pp-F (BS-2)** | TGTTTATGAATTGTGTTTATTTTGTAAGTA |
| ***Rhox5*-Pp-R (BS-2)** | TCTAAACTTAAACCCCTAATATCCC |
| ***Rhox5*-TSS-F (BS-3)** | GGGGATATTAGGGGTTTAAGTTTAG |
| ***Rhox5*-TSS-R (BS-3)** | CAAAACTCATTTACTTCCCAAAAAC |
| **ChIP assays** |  |
| ***Rhox5*-1-F (ChIP-1)** | CAGCCACTCATGCTTCTTCA |
| ***Rhox5*-1-R (ChIP-1)** | TCACCAGGACAAGAGGAACC |
| ***Rhox5*-2-F (ChIP-2)** | GCTGTAACTGGGCACCCTAA |
| ***Rhox5*-2-R (ChIP-2)** | CAGCCCTAAGCTGGTCTTTG |
